# Disease-Related Protein Variants of the Highly Conserved Enzyme PAPSS2 Show Marginal Stability and Aggregation in Cells

**DOI:** 10.3389/fmolb.2022.860387

**Published:** 2022-04-08

**Authors:** Oliver Brylski, Puja Shrestha, Philip J. House, Patricia Gnutt, Jonathan Wolf Mueller, Simon Ebbinghaus

**Affiliations:** ^1^ Institute of Physical and Theoretical Chemistry, TU Braunschweig, Braunschweig, Germany; ^2^ Institute of Physical Chemistry II, Ruhr University, Bochum, Germany; ^3^ Institute of Metabolism and Systems Research (IMSR), University of Birmingham, Birmingham, United Kingdom; ^4^ Centre for Endocrinology, Diabetes and Metabolism (CEDAM), Birmingham Health Partners, Birmingham, United Kingdom

**Keywords:** PAPS synthase, sulfation pathways, in-cell spectroscopy, protein folding, stability and aggregation

## Abstract

Cellular sulfation pathways rely on the activated sulfate 3′-phosphoadenosine-5′-phosphosulfate (PAPS). In humans, PAPS is exclusively provided by the two PAPS synthases PAPSS1 and PAPSS2. Mutations found in the PAPSS2 gene result in severe disease states such as bone dysplasia, androgen excess and polycystic ovary syndrome. The APS kinase domain of PAPSS2 catalyzes the rate-limiting step in PAPS biosynthesis. In this study, we show that clinically described disease mutations located in the naturally fragile APS kinase domain are associated either with its destabilization and aggregation or its deactivation. Our findings provide novel insights into possible molecular mechanisms that could give rise to disease phenotypes associated with sulfation pathway genes.

## Introduction

Sulfation is a highly important biological process where a sulfate moiety from the activated sulfate donor 3′-phosphoadenosine-5′-phosphosulfate (PAPS) is transferred onto acceptor molecules. Adding the negatively charged sulfate group to a hydroxyl-group induces significant changes in the chemical properties of the acceptor molecule with a major impact on their function. It is the sheer variety of sulfated metabolites that makes sulfation impactful on numerous biological systems. Sulfotransferases use activated sulfate to modify proteins, glycans and other biomolecules like steroid hormones (Klassen and Boles, 1997; Strott, 2002; Mueller et al., 2015).

Active PAPS synthase enzymes generate active sulfate in the form of PAPS. In humans, there are two isoforms PAPSS1 and PAPSS2 (van den Boom et al., 2012). Nevertheless, disease-related protein variants have been exclusively reported for PAPSS2.

In a variety of human genetics studies, a total of 65 individuals with various inactivating alleles of the human PAPSS2 gene have been described (Ahmad et al., 1998; Haque et al., 1998; Noordam et al., 2009; Miyake et al., 2012; Iida et al., 2013; Tüysüz et al., 2013; Oostdijk et al., 2015; Handa et al., 2016; Bownass et al., 2019; Eltan et al., 2019; Perez-Garcia et al., 2021). These have been analyzed and summarized recently (Baranowski et al., 2018; Brylski et al., 2019; Paganini et al., 2020). An additional PAPSS2 variant is known for brachymorphic mice (Kurima et al., 1998).

Recently, several studies investigated the essential steps in sulfation pathways, whose malfunction is correlated with disease symptoms (Mueller et al., 2015; Foster and Mueller, 2018). Among these symptoms are bone and cartilage dysplasia (Oostdijk et al., 2015), as well as androgen excess and polycystic ovary syndrome (PCOS) (Noordam et al., 2009; Oostdijk et al., 2015), all caused by point-mutations in the *PAPSS2* gene encoding for the PAPS synthase 2 enzyme. These point mutations diminish the enzyme activity and they are mainly located within the kinase domain of PAPSS2 (Kurima et al., 1998; Noordam et al., 2009; Iida et al., 2013).

It is evident that PAPSS2 plays a vital role in skeletal development as well as steroid hormones regulation (Kurima et al., 1998; Noordam et al., 2009; Iida et al., 2013). Kurima and coworkers linked the mutation G78R within the nucleotide kinase domain of the PAPSS2 isoform, with the bone phenotype seen in the brachymorphic mouse (*bm*) (Kurima et al., 1998). G78R is located in the adenosine-5′-phosphosulfate (APS) kinase domain, close to the ligand-binding site. The mutation causes catalytic inactivation and hence lowered intracellular PAPS availability. As a consequence, *bm* mice show reduced postnatal growth that was ascribed to under-sulfation of the extracellular matrix; they also show abnormal hepatic detoxification and prolonged bleeding times (Kurima et al., 1998). One of the most prominent roles of sulfation is the modification of glycosaminoglycans (GAGs) by Golgi-residing carbohydrate sulfotransferases (Gesteira et al., 2021). Sulfated GAGs play a vital role in cell signaling to regulate many biochemical processes like cell growth and proliferation, promotion of cell adhesion, anticoagulation and wound repair (Raman et al., 2005; Prydz, 2015). In brachymorphic cartilage, GAGs are found at normal level but significantly under-sulfated, affecting the formation of connective tissue, such as, cartilage (Kurima et al., 1998; Cho et al., 2004).

More recently, Noordam and coworkers reported a case study of a girl with premature pubarche, hyperandrogenic anovulation, very low level of dehydro-epiandrosterone sulfate (DHEAS) and high level of androgen. The steroid sulfation defect of this patient was associated with a T48R mutation found in the APS kinase domain. Due to this mutation, PAPS synthesis is affected, leading to incompetent DHEA inactivation, with the latter resulting in increased levels of androgens causing PCOS-like phenotypes (Noordam et al., 2009). In 2013, Iida and coworkers reported more PAPSS2 mutations (C43Y, L76Q, E183K, V540D) out of which three, C43Y, L76Q and E183K, were found in the APS kinase domain. C43Y and L76Q cause loss of function leading to brachyolmia and abnormal androgen metabolism (Iida et al., 2013).

Eukaryotic cells express another PAPS synthase gene, PAPSS1, that shares 78% identity at the level of amino acid sequence (van den Boom et al., 2012). However, this protein isoform cannot compensate for the loss of the other (Mueller et al., 2018). This lack of compensation raises the question of whether the two isoforms impact differently on subsets of sulfation pathways. Subcellular localization sequences (Schröder et al., 2012) were identified in both PAPS synthases and dimer formation (Sekulic et al., 2007; Grum et al., 2010; Brylski et al., 2019) was observed, both features proposed to be crucial for proper localization and activity of the enzyme. In addition to these physiological aspects, *in vitro* biophysical studies focused on the stability of PAPS synthases revealed that isoforms of this enzyme are only marginally stable as recombinant proteins (van den Boom et al., 2012). However, PAPS synthase proteins can be stabilized by preferential binding of their substrates to the APS kinase domain, namely PAPS, adenosine diphosphate (ADP) and APS (van den Boom et al., 2012; Mueller and Shafqat, 2013).

Using a recently developed folding sensor of the APS kinase domain of the human PAPS synthase PAPSS2 (Brylski et al., 2021), we investigate how clinically reported single-point-mutations change the in-cell stability of the APS kinase domain and if destabilization could lead to aggregation and thus loss of metabolic activity.

## Materials and Methods

### Construction of PAPSS2 and APSK37 Variants

The pEGFP-C1-PAPSS2 plasmid encoding human full-length PAPSS2b C-terminally fused to an EGFP fluorescent protein (Schröder et al., 2012), was used for DpnI-based site-directed mutagenesis and subsequently for cell counting experiments. To generate the APSK37 sensor, the APS kinase domain of PAPSS2 was PCR-subcloned into a modified pDream2.1 vector with an N-terminal AcGFP1 and a C-terminal mCherry (Ebbinghaus et al., 2010). The APSK enzyme was truncated between the two isoleucine residues I220 and I221, within the flexible linker that connects the kinase and the sulfurylase domains (Harjes et al., 2005). Furthermore, the flexible and disordered N-terminal region, which is known to assist in the dimerization of the protein (Sekulic et al., 2007; Grum et al., 2010), was truncated by 37 amino acids (Δ37). Further, DpnI-based site-directed mutagenesis was used to introduce different disease related point mutations (G78R, L76Q, C43Y and T48R). All constructs were verified by Sanger DNA sequencing.

### Cell Culture and Plasmid Transfection

HeLa cells were grown at 5% CO_2_ at 37°C in DMEM supplemented with 10% FBS, 100 U/ml penicillin and 0.1 mg/ml streptomycin. Cells were passaged at a 1:4 or 1:6 ratios at 80–90% confluence, using trypsin digestion. For transfection, cells were seeded in six-well plates (Sarstedt). Using Lipofectamine 3000 (Thermo Fisher), cells were transfected according to the manufacturers protocol. Concisely, a mixture of 125 µl Opti-MEM (Thermo Fisher) with 2 µg of the respective plasmid DNA and 4 µl P3000 reagents was prepared. After 5 min of incubation, the mixture was transferred to another solution containing 125 µl Opti-MEM supplemented with 4 µl Lipofectamine3000 reagent. Cells were incubated for 6 h after the addition of transfection mixture to the cellular growth medium at 5% CO_2_, 37°C. The cells were passaged using trypsin digestion and seeded in 35 mm glass bottom dishes (Fluorodish, World Precision Instruments). Cells were grown for 2 days at standard cell culture conditions before imaging.

### Sample Preparation

Fast Relaxation Imaging (FReI) was performed with transfected cells grown on 35 mm glass bottom dishes (Fluorodish, World Precision Instruments). Cells were washed with Dulbecco’s Phosphate Buffered Saline (DPBS) (Sigma-Aldrich) after removing the growth medium. 30 µL Leibovitz’s L15 medium supplemented with 30% FBS were sealed between a glass cover slip (Menzel #1.0) with a 120 µm thick imaging spacer (Sigma-Aldrich) and a glass bottom dish with cells.

### Fast Relaxation Imaging Measurements

FReI is a combination of wide field fluorescence microscopy with millisecond temperature jumps induced by an IR diode laser (m2k-Laser, 2200 nm). The technique was previously described (Ebbinghaus et al., 2010; Gnutt et al., 2019a). Shortly, fluorescent light was split by a dichroic beam splitter into donor and acceptor signal that was recorded using CCD cameras while the sample is rapidly heated by an IR laser. The temperature sensitive dye Rhodamine B (Sigma Aldrich) was used for the calibration of temperature jumps (Vöpel et al., 2015; Gao et al., 2016; Büning et al., 2017). The heat profile used in this study showed an average temperature increase of 2.2°C per jump at intervals of 50 s, covering a range from 23.0 to 58.2°C in 16 steps. Image acquisition was performed at one frame per second (fps) with LED exposure times typically between 50 and 200 ns. Data was recorded using AxioVision software and the images were processed and analyzed using ImageJ (National Institute of Health, United States) and further evaluated using self-written MatLab (Mathworks) codes and GraphPad Prism 6 (GraphPad).

For data analysis, fluorescence intensities were averaged throughout the cytoplasmic region for each channel individually (Dhar et al., 2011). Further, background subtraction was performed for the individual channels and the ratio of the donor and acceptor channel (D/A) calculated. The changes of D/A ratio upon temperature jump yield information about the associated conformational change. An increase in D/A refers a decrease in FRET that may be attributed to protein unfolding. To analyze the kinetics of protein unfolding, the individual channel intensities were used as D-αA according to (Dhar et al., 2011). To determine the melting point (T_M_) of the protein, the thermodynamic model introduced as *Better thermodynamics from kinetics* (Girdhar et al., 2011) was used:

$$D-\alpha A\left(T\right)=\frac{-\delta g_{1}\Delta T\cdot T_{m}}{R{\left(T-\Delta T/2\right)}^{2}}\cdot \left(A_{0}+m_{A}\left(T-T_{m}\right)\right)\cdot \frac{\text{exp}\left(-\delta g_{1}\left(T-\left(\Delta T/2\right)-T_{m}\right)\cdot {\left(R\left(T-\Delta T/2\right)\right)}^{-1}\right)}{{\left(1+\text{exp}\left(-\delta g_{1}\left(T-\left(\Delta T/2\right)-T_{m}\right)\cdot {\left(R\left(T-\Delta T/2\right)\right)}^{-1}\right)\right)}^{2}}$$

Where, ∂g1 is pre-factor of the linear Taylor approximation of the two-state populations. ΔT is the amplitude of the temperature jump (set to 2.2°C) and A_0_ and m_A_ are fitting parameters of the underlying baseline (with m_A_ set to 0).

### HEK293 Cell Culture and Wide Field Microscopy

HEK293 cells were cultured in DMEM with high glucose (Gibco, United Kingdom), supplemented with 10% fetal FBS and penicillin/streptomycin at 1%. Cells were passaged at 80–90% confluence, using trypsin digestion. Regular checks ensured that all cells were mycoplasma-free. Cells were seeded 1:8 or 1:10 in culture flasks or maintaining stocks or at 200,000 cells per well into six-well plates with microscopic slides in them. Transfection of these HEK293 cells 24 h after seeding on cover slips was performed using the XtremeGENE HP DNA transfection reagent (Roche, United Kingdom), according to manufacturer’s instructions. Cells were left growing for 24 or 48 h, then washed with ice-cold PBS and incubated with ice-cold methanol, followed by three further washing steps using PBS. Finally, cover slips with cells were mounted on microscopic slides, using fluorescence mounting media, and fixed with nail varnish. The slides were anonymized to enable blind, non-biased analysis. The slides were then viewed under a wide-field fluorescent microscope and scored at least three different sections using a ×20 objective. Cells were imaged with a ×20 objective. The number of speckles per cell was scored in large numbers of cells, in a blinded fashion. As a control, fluorescence intensity was ranked as well (low/medium/high). No correlation was found between fluorescence intensity and number of speckles, suggesting that protein over-expression levels were not linked to the observed patterns of speckles. Significant changes of population of non-speckled cells have been determined using two-way ANOVA with a *post-hoc* Holm-Sidak’s test correcting for multiple comparisons.

## Results

### Mutations in APSK37 Reveal Distinct Folding Stabilities

We analyzed the effect of disease-related mutations on the folding stability and aggregation of the APS kinase domain of the bifunctional PAPSS2 protein, using our recently established APSK folding sensor (APSK37) (Brylski et al., 2021). The sensor reports intramolecular FRET between the N-terminal AcGFP1 and the C-terminal mCherry fusion proteins (*see* Materials and Methods for details).

**FIGURE 1:**
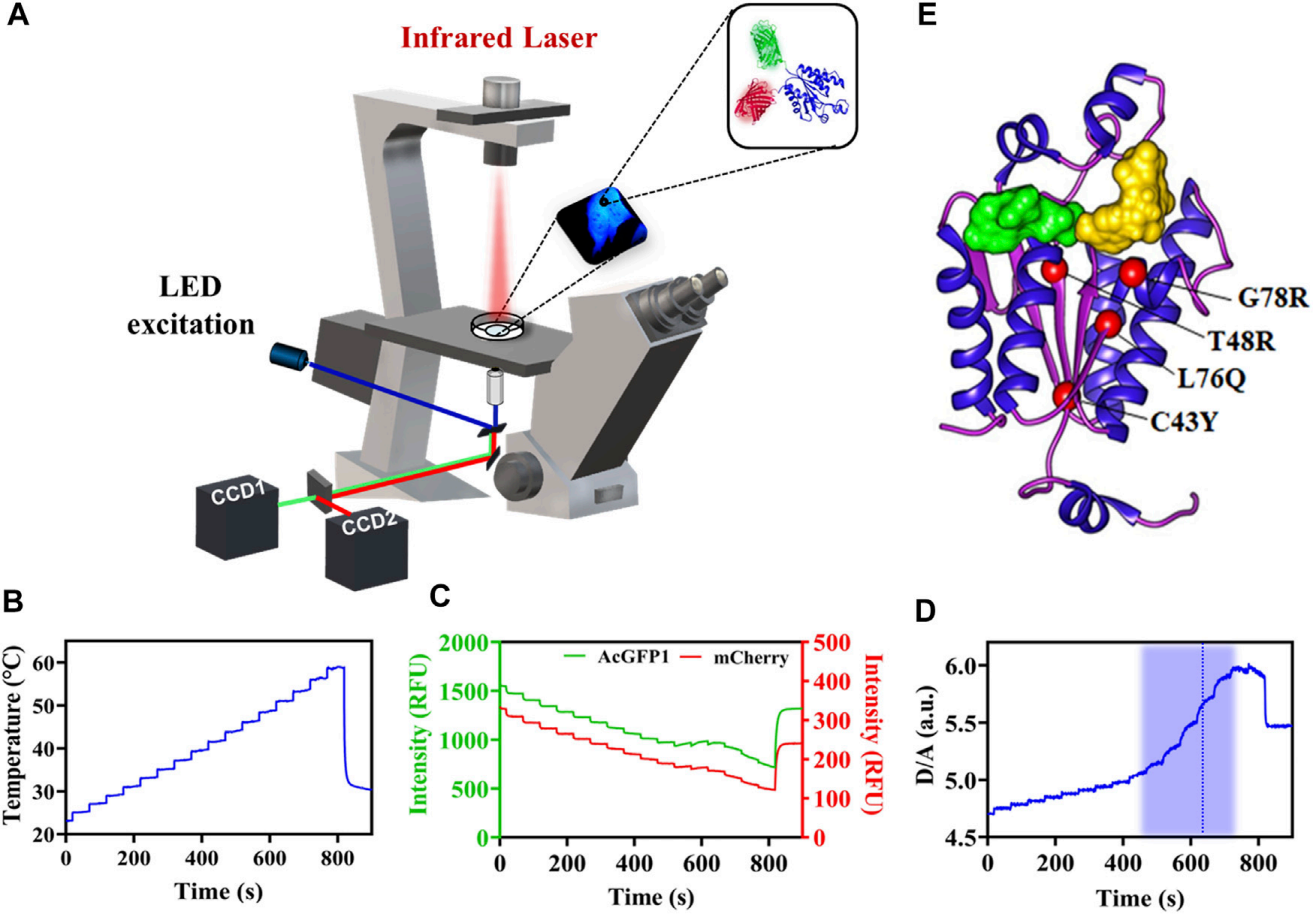
In-cell thermal unfolding of APSK37 wt using Fast Relaxation Imaging. **(A)** Schematic representation of the Fast Relaxation Imaging setup. **(B)** Induced temperature profile calculated by the calibration procedure published in Büning et al., 2017. **(C)** Change in fluorescence according to the temperature profile in B of the APSK37 AcGFP1 FRET donor (D) and the mCherry FRET acceptor **(A)**. **(D)** D/A ratio calculated from the intensity data in **(C)**. The thermal unfolding region is shaded in blue and the calculated T_M_ is displayed by a dash blue line. **(E)** Crystal structure of the APSK domain of PAPSS2 (PDB:2AX4). Studied mutations are shown as red spheres. ADP/ATP (green) and APS/PAPS (yellow) binding sites are indicated by the substrates surface representation.

We analyzed PAPS synthase disease point mutations located in the APS kinase domain to understand whether the phenotypes seen clinically correlate with misfolded, destabilized or inactive protein. Therefore, we created the variants G78R, L76Q, T48R and C43Y within APSK37 (Oostdijk et al., 2015), expressed them in HeLa cells and studied their in-cell protein stability in comparison to the wt protein using Fast Relaxation Imaging (FReI). In FReI, a rapid perturbation of temperature is applied by absorption of infrared light (IR) by the sample (Ebbinghaus et al., 2010; Vöpel et al., 2017; Gnutt et al., 2019b) (Figures 1A,B). Dual-color imaging allows to measure changes in donor-to-acceptor intensity ratio (D/A) (Figure 1C) that display unfolding kinetics and thermodynamics of the protein in the cell (*see* Materials and Methods for details) (Figure 1D).

A structural analysis of the surface exposure of the disease-related mutants revealed that C43Y, T48R and L76Q are deeply buried in the protein core (solvent accessible surface area (SASA) ≤ 1 Å^2^) compared to G78R (Kabsch and Sander, 1983; Brylski et al., 2019); all of them located in close proximity in the central beta-sheet of the APSK (Figure 1E).

**FIGURE 2:**
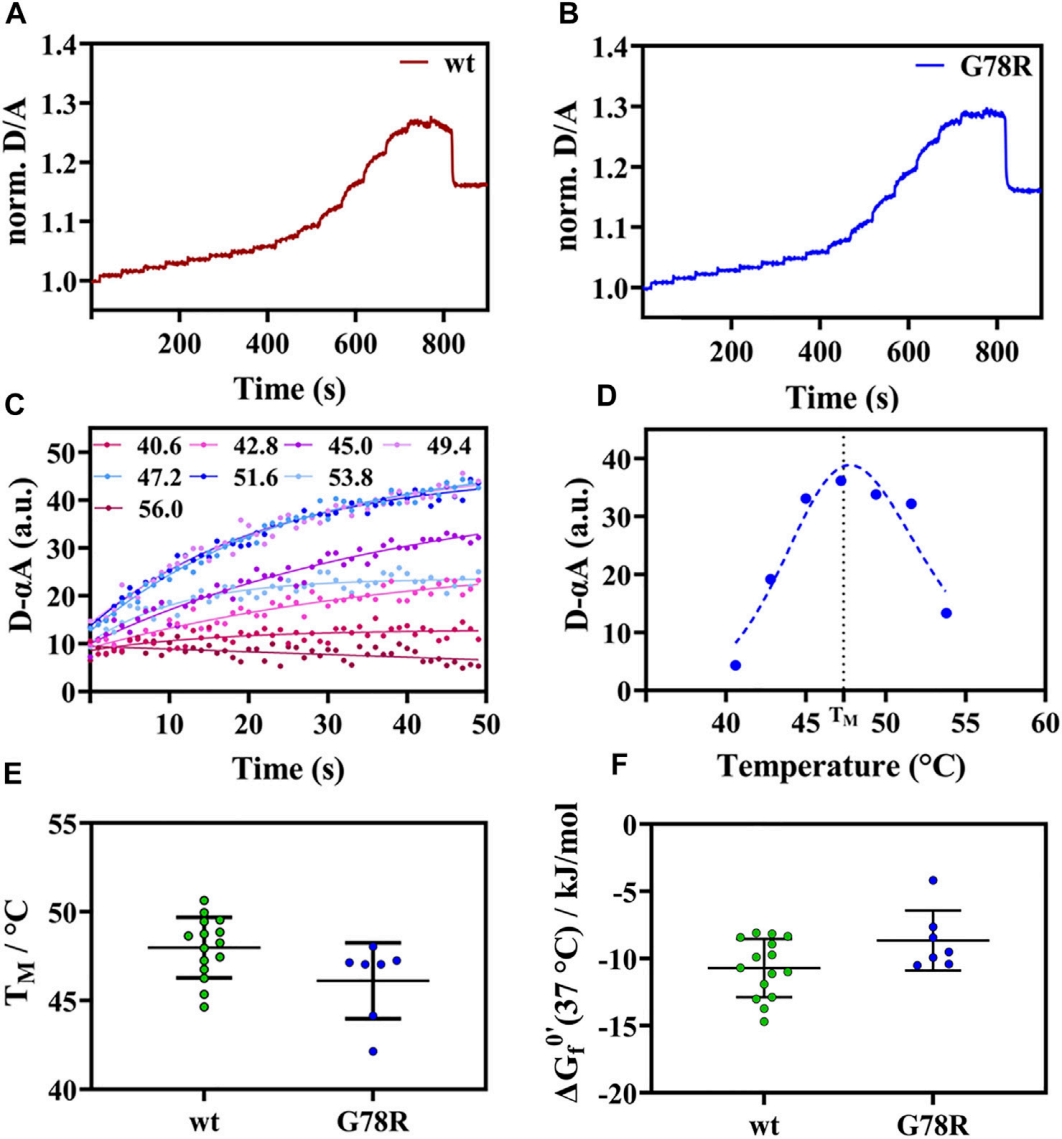
Thermal unfolding curve APSK37 wt and mutants (G78R). **(A)** Exemplary temperature induced thermal unfolding curves of wt (data shown from (Brylski et al., 2021)). **(B)** Exemplary temperature induced thermal unfolding curves of G78R. **(C)** Exponential unfolding curves of single temperature jumps from panel **(B)** showing the relaxations kinetics at the respective temperatures. **(D)** Kinetic amplitudes as a function of temperature to determine the T_m_ (dashed line). **(E)** Thermal stability comparison of the mutant G78R with APSK37 wt: Melting points of APSK37 (green) and melting point of G78R (blue) derived from FReI measurrement showing average ± s.d. **(F)** Folding free energy ∆G_f_^0’^ for both APSK37 wt and G78R mutant with mean ± s.d. There are no statistically significant differences between wt and G78R. Significance were tested *via* one-way ANOVA with a *post-hoc* Holm-Sidak test correcting for multiple comparisons (no significant changes observed).

For APSK37 wt, we observed an increase of the normalized D/A ratio upon IR-laser heating and decrease after returning to the starting temperature (Figure 2A). This behavior is also evident for G78R mutant (Figure 2B), which exhibits an apparent two-state folding behavior as the respective unfolding kinetics can be fitted by a single exponential function. Plotting and fitting the respective amplitudes against temperature (Figures 2C,D) allowed the determination of a T_M_ = 46.1 ± 2.1°C which is similar to wt (48.0 ± 1.7°C) (Figure 2E). Additionally, no significant differences were found with respect to the modified standard state free energies of folding ΔG_f_^0’^, suggesting that this mutation does not affect the stability of the protein (Figure 2F).

**FIGURE 3:**
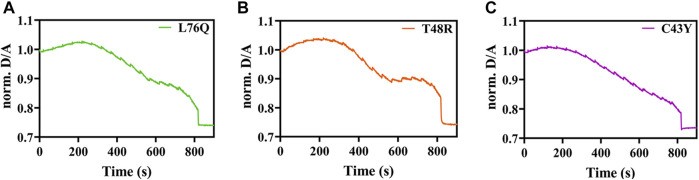
Exemplary temperature-induced thermal unfolding curves of the L76Q, T48RM, and C43Y mutants are visible in Figures 3A–C, respectively.

For the mutations C43Y, T48R and L76Q, we did not detect any unfolding transitions, impeding the determination of T_M_. We rather observed a strong decrease in the D/A ratio (Figures 3A–C) that can be attributed to an increase in FRET by intermolecular energy transfer due to self-association (Ebbinghaus et al., 2010; Büning et al., 2017).

### Self-Association and Aggregation of C43Y, T48R and L76Q

**FIGURE 4:**
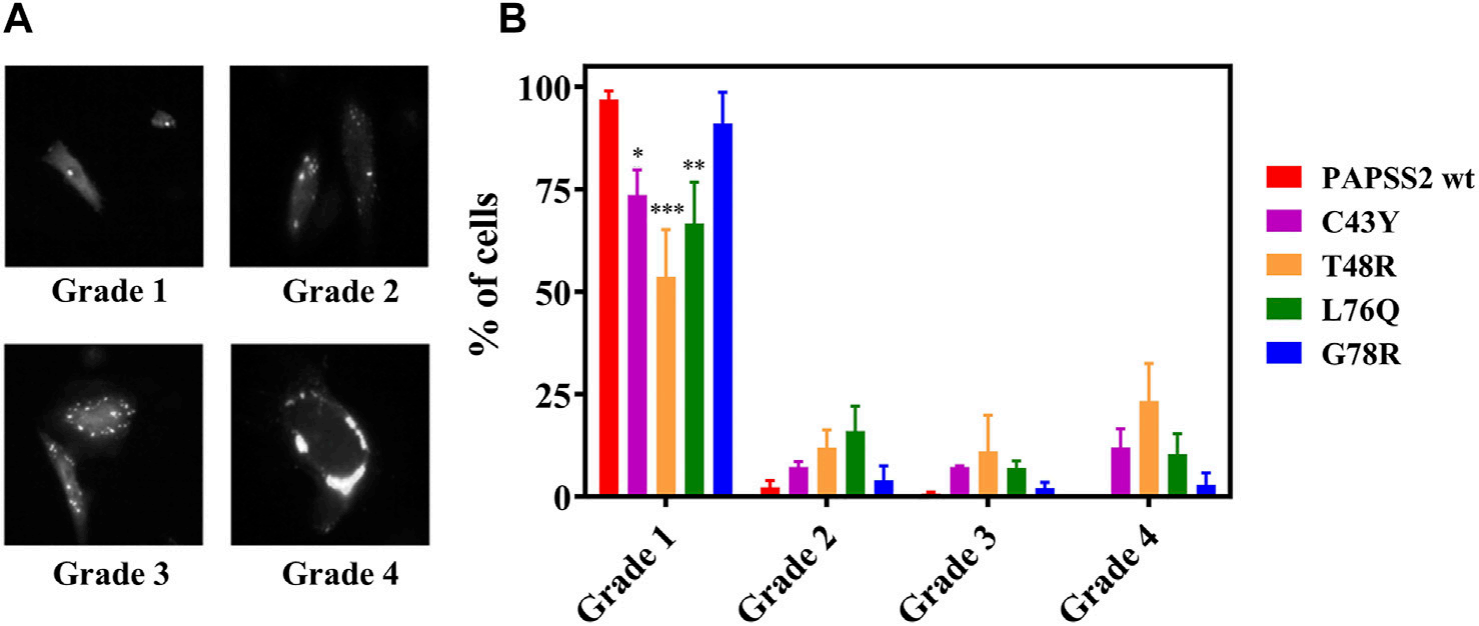
Expression and distribution of recombinant human PAPSS in HEK293 cells. **(A)** Exemplary fluorescence image of HEK293 cells showing fluorescence spots classified according to three categories. **(B)** Even distribution; no speckles: grade 1, 1–3 speckles: grade 2, 4–10 speckles: grade 3 and more than 10 speckles or large clumps: grade 4. Data is presented as average ± s.e.m. Cells were counted from four different slides (*N* = 4) with *n* > 200 cells in total for each protein variant. Asterisks indicate significant differences of the fraction of cells showing no speckles compared to PAPSS2 wt (*p < 0.05, ***p < 0.001). Additional statistical significance within grade 1 was found between G78R and T48R(***) or G78R and L76Q(*).

We then investigated if the self-association events observed in FReI result in the formation of microscopically visible aggregates. We monitored their formation by wide-field fluorescence microscopy of EGFP-labeled full-length human PAPS synthase 2 carrying the disease mutants, expressed in HEK293 cells along with PAPSS 2 wt proteins. The cellular distribution pattern of the protein and degree of aggregate formation was scored by classifying individual cells according to the number of speckles that were visible inside each cell (Figure 4A). Figure 4B illustrates the cellular distribution pattern of the protein and the degree of aggregate formation for different mutants compared to PAPSS2 wt. The mutants C43Y, T48R and L76Q caused a higher number of aggregates compared to PAPSS2 wt. The level of aggregate formation of the G78R mutation is not significantly different from wt. Thus, the results show that the self-association of the C43Y, T48R and L76Q APSK37 proteins measured by FReI is in accordance with protein aggregation of the respective mutants in the full-length PAPSS2 protein. On the other hand, the G78R mutant is stable and does not lead to aggregation both in APSK37 and PAPSS2 proteins.

## Discussion

Generally, proteins with disease-related mutations either show a loss of catalytic function, a gain of toxic function (Winklhofer et al., 2008; De Baets et al., 2015) or a significant loss in stability of the protein leading to misfolding and aggregation (Waters, 2001; Denny et al., 2013; Gandhi et al., 2019). For PAPSS2, many studies have shown that disease-related gene defects cause different forms of bone and cartilage malformation (Kurima et al., 1998; Iida et al., 2013), as a consequence of under-sulfation of extracellular matrix. Dysregulation of steroid metabolism causing an increase in androgen activation is further associated with diseases like PCOS and premature pubarche (Noordam et al., 2009).

The results of this study show that PAPSS2 disease-related mutations cause a destabilization and aggregation of the enzyme in cellular environments for the mutants C43Y, T48R, L76Q. The G78R mutation however shows a folding stability that is comparable to wt, preventing aggregation. Regarding catalysis, Kurima and coworkers have reported that the G78R variant has very little residual APS kinase activity, but the ATP sulfurylase activity was comparable to wt (Kurima et al., 1998). Conformational changes of the APS binding site upon mutation, modifying the interaction between the ATP γ-phosphate group, the magnesium ion and the DGDN-loop can be a potential reason for the catalytic inhibition APS kinase (Kurima et al., 1998). The mutation may not disrupt the native fold, however, a catalytic conversion, for example due to a loss in flexibility within the DGDN-loop, may not be possible anymore.

The mutations C43Y, T48R, and L76Q destabilize APSK37 and lead to aggregation of both APSK37 and full-length PAPS synthase. All three mutations reside in the central β-sheet region of the protein (Figure 1E), suggesting that this is a sensitive region that maintains the native fold and prevents self-association and aggregation. Changes in the protein’s native structure, protein-protein interactions and many other sequential and parallel events can lead to misfolded/unfolded conformations, resulting in aggregation. Protein aggregation is often linked with various pathologies, including neurodegenerative diseases, such as Alzheimer’s, Parkinson’s and Huntington’s. These disease-related aggregates are generally sub-divided into loss-of-function and gain-of- toxic function effects (Ross and Poirier, 2004; Wang, 2005; Soto and Pritzkow, 2018). Indeed, the three above-mentioned PAPSS mutations were previously classified as “missense mutants,” causing its loss of function (Noordam et al., 2009; Iida et al., 2013). Missense mutations in nuclear deubiquitinase BAP1 were previously shown to induce destabilization and aggregation of this enzyme, with the latter being suggested as the main cause of its functional loss (Bhattacharya et al., 2016). In fact, this hypothesis is further supported by our previous studies (Brylski et al., 2021) using an alanine scanning mutagenesis of the substrate binding site of APSK37 in HeLa cells. The results revealed a large range of different in-cell stabilities for the single point mutations (ΔG_f_^0^ = −10.7 to +13.8 kJ/mol).

Our results suggest two distinct possible disease mechanisms, one related to misfolding and aggregation, and the other one related to inhibition of catalytic function. However, whether these processes are causal for the different pathologies needs to be elucidated in future studies.

## Conclusion

So far, many sulfotransferase-related mutations are known to be associated with the sulfation pathway but a lot less is reported for PAPSS. Our results report that PAPSS2 disease-related mutations cause misfolding and aggregation (L76Q, T48R, and C43Y), and inhibition of the catalytic function (G78R). Even though our study showed that the three missense mutants (L76Q, T48R and C43Y) lead to aggregation, the molecular details of this process remains to be explored, particularly putative cytotoxic effects of amyloid formation. Therapeutic approaches against the rare diseases that are associated with these mutations may thus be different, encompassing supplementation of lacking compounds (Klinge et al., 2018) or inhibitors to reduce aggregation (Vöpel et al., 2017).

## Data Availability

The original contributions presented in the study are included in the article/Supplementary Material, further inquiries can be directed to the corresponding authors.
